# Anniversary

**Published:** 1890-07

**Authors:** 


### Editorial.

### ANNIVERSARY.

Just one year ago the JOURNAL passed into the hands of its present management (with additional changes noted in January issue), and while we do not wish to boast, we feel sure our friends will be glad to know that it has been one of the most prosperous years in the history of the JOURNAL. During that time we have more than doubled our circulation, and largely increased our advertising patronage. And this success is the more gratifying to us because it has been honestly attained. We have not and will not claim a larger circulation than we actually have. The tone and character of the reading matter with which we present our subscribers—while not all that we might have wished for—it has been, and shall be, our constant endeavor to elevate. There are, however, certain features of the JOURNAL that have been blemishes. The one that has been especially distasteful to us, as well as to our readers, is the continued insertion of inset advertising pages. We expect to remedy this just as fast as our present contracts expire. There are other things which our readers alone can remedy. For instance, we would like to publish each month a number of short practical articles from general practitioners reporting their interesting cases, etc. But it is impossible to publish such articles when we cannot get them.

In conclusion we wish to thank our friends for their liberal patronage, and to assure them that it shall be our constant endeavor to better deserve it.

